# Redox-Modulating Capacity and Antineoplastic Activity of Wastewater Obtained from the Distillation of the Essential Oils of Four Bulgarian Oil-Bearing Roses

**DOI:** 10.3390/antiox10101615

**Published:** 2021-10-14

**Authors:** Almira Georgieva, Yana Ilieva, Zlatina Kokanova-Nedialkova, Maya Margaritova Zaharieva, Paraskev Nedialkov, Ana Dobreva, Alexander Kroumov, Hristo Najdenski, Milka Mileva

**Affiliations:** 1 The Stephan Angeloff Institute of Microbiology, Bulgarian Academy of Sciences, 26 Acad. G. Bonchev str., 1113 Sofia, Bulgaria; al.georgieva@inb.bas.bg (A.G.); ilievayana@microbio.bas.bg (Y.I.); zaharieva26@yahoo.com (M.M.Z.); adkrumov@gmail.com (A.K.); hnajdenski@abv.bg (H.N.); 2 Institute of Neurobiology, Bulgarian Academy of Sciences, 23 Acad. G. Bonchev str., 1113 Sofia, Bulgaria; 3 Faculty of Pharmacy, Medical University of Sofia, 2 Dunav str., 1000 Sofia, Bulgaria; zlatina.kokanova@pharmfac.musofia.bg (Z.K.-N.); pnediakov@pharmfac.musofia.bg (P.N.); 4 Department of Aromatic and Medicinal Plants, Institute for Roses and Aromatic Plants, 49 Osvobojdenie Blvd, 6100 Kazanlak, Bulgaria; anadobreva@abv.bg

**Keywords:** wastewater, UHPLC-HRMS analysis, antineoplastic activity, apoptosis induction, redox potential, nonlinear modeling

## Abstract

The wastewater from the distillation of rose oils is discharged directly into the soil because it has a limited potential for future applications. The aim of the present study was to determine in vitro the chromatographic profile, redox-modulating capacity, and antineoplastic activity of wastewater obtained by distillation of essential oils from the Bulgarian *Rosa alba* L., *Rosa damascena* Mill., *Rosa gallica* L., and *Rosa centifolia* L. We applied UHPLC-HRMS for chromatographic analysis of rose wastewaters, studied their metal-chelating and Fe(III)-reducing ability, and performed MTT assay for the evaluation of cytotoxic potential against three tumorigenic (HEPG2—hepatocellular adenocarcinoma, A-375—malignant melanoma, A-431—non-melanoma epidermoid squamous skin carcinoma) and one non-tumorigenic human cell lines (HaCaT—immortalized keratinocytes). The median inhibitory concentrations (IC_50_) were calculated with nonlinear modeling using the MAPLE^®^ platform. The potential of the wastewaters to induce apoptosis was also examined. Mono-, di-, and acylated glycosides of quercetin and kaempferol, ellagic acid and its derivatives as main chemical components, and gallic acid and its derivatives—such as catechin and epicatechin—were identified. The redox-modulating capacity of the samples (TPTZ test) showed that all four wastewaters exhibited the properties of excellent heavy metal cleaners, but did not exert very strong cytotoxic effects. The lowest IC_50_ rate was provided in wastewater from *R. centifolia* (34–35 µg/mL of gallic acid equivalents after a 72 h period for all cell lines). At 24 and 48 hours, the most resistant cell line was HEPG2, followed by HaCaT. After 72 h of exposure, the IC_50_ values were similar for tumor and normal cells. Still, *R. damascena* had a selectivity index over 2.0 regarding A-431 non-melanoma skin cancer cells, showing a good toxicological safety profile in addition to moderate activity—IC_50_ of 35 µg/mL polyphenols. The obtained results related to wastewaters acquired after the distillation of essential oils from the Bulgarian *R. alba*, *R. damascena*, *R. gallica*, and *R. centifolia* direct our attention to further studies for in-depth elucidation of their application as detoxifying agents under oxidative damage conditions in other experimental datasets.

## 1. Introduction

It is very interesting that plants from the Rosaceae family have a great influence on world history and are among the most valuable oil-bearing plants. For centuries, different civilizations have used them to condiment the foods of kings and nobles, and applied them as embalming preservatives, perfumes, cosmetics, and medicines in various regions of the world [1,2,3,4]. In general, these plants have formed the basis of traditional medicine, and some of their derived substances have been utilized to treat different human diseases. Representatives of the genus *Rosa* deserve special attention; they are one of the most important groups of ornamental plants, and seen as a sign of inspiration, purity, love, happiness, and beauty [2,4].

At the present time, the biggest producers of roses are countries such as Turkey, China, the countries of the former Soviet Union, Egypt, Morocco, and Bulgaria. In Bulgaria, the cultivation and processing of roses is a tradition and livelihood of a large part of the population and is very important for the agricultural economy of the country. *Rosa damascena* Mill., *Rosa alba* L., *Rosa gallica* L., and *Rosa centifolia* L. are the main species grown [5,6].

In Bulgaria, the most popular method for the production of rose oil is a classical method of water–steam distillation, which leaves a water fraction as a remnant of the technological process [5,7]. It can be said that rose oil is more expensive than gold—more than 40 kg of rose inflorescences are used to produce one gram of oil [8].

The low yield of essential oil leads to the release of bulky waste—(1) used rose petals as a solid residue, as well as (2) wastewater (liquid residue) [9].

In this sense, the wastewaters (WWs) after rose oil distillation represent a serious environmental problem as pollutants, because they are discarded into drainage systems and rivers [4].

Already, at the beginning of this century, there was talk of recycling waste products from various industries. The valorization of plant materials and reuse of the potentially bioactive ingredients of these materials became the subject of numerous research investigations. Many novel approaches have been studied, including the extraction of polysaccharides from the biomass and the introduction of integrated methods for the more complete valorization of the rose waste byproducts. Due to insufficient data on their composition and biological activities, most of the methods for rose waste valorization still remain confined to the laboratory scale [9].

Rose WW, as a byproduct of hydrodistillation, is rich in water-soluble compounds such as polyphenols, glycosides, tannins, etc., so it can be used as a good, natural, inexpensive source of biologically active compounds and high-value products after more phytochemical, in vitro, and in vivo studies [4,10].

These compounds are the subject of a rather scarce but growing number of studies in terms of their biological potential. Sabahi et al. (2020) applied a cytotoxicity assay on HEP-G2 cells (human liver cancer cell line) exposed to different concentrations of *R. damascena* polyphenol-enriched fraction of WW for 24 and 48 h. Significant toxicity at a concentration of 100 μg/mL and higher (*p* < 0.001) was reported in this cell line [10].

However, these kinds of results need to be validated in in vivo settings [11]. Different components of *R. alba* have been subject to various studies. Naikwade et al. (2009) reported a learning and memory-enhancing activity of *R. alba*; The authors indicated that aqueous extract of *R. alba* L. might be a useful memory-restorative agent for treating cognitive disorders such as Alzheimer’s disease [12]. Polyphenols are among the most common secondary metabolites in the plant kingdom, involved in plants’ pigmentation and their antioxidant defense system against UV light [13,14]. Chemically, polyphenols are largely planar molecules, and can be divided into several classes, e.g., phenolic acids, flavonoids, isoflavonoids, proanthocyanidins, anthocyanins, stilbenes, and lignans [15]. Polyphenols are known to have a wide range of biochemical activities, such as free radical scavenging and metal chelating, antioxidant, anticancer, anti-inflammatory, and antimutagenic activities, etc. (Table 1, also See Table S1 in Supplementary Materials). 

Their activities are based on their ability to be excellent donors of hydrogen, which is accepted by reactive radicals to yield much less active radical and non-radical species [16]. In cells under normal physiological conditions, there is a balance between the rate of generation of ROS and their utilization by the cell’s antioxidants [17,18]. The redox state of a cell determines its cellular functioning, and is usually kept within a narrow range under normal conditions [17]. The fine balance between the useful and detrimental effects of ROS is due to the metabolic reactions that use oxygen and constitute a very important part of protecting living organisms and maintaining “redox homeostasis” by controlling the redox regulation in vivo [19].

In the scientific literature, some approaches and suggestions for rose waste utilization have been described and sought, as well as suggestions for the reuse of the WWs, and the compounds with potentially beneficial effects contained therein. One such study provides evidence that phenolic compounds in water byproducts obtained after hydrodistillation of Taif rose (*Rosa damascena trigintipetala* Dieck) showed antioxidant activity [20]. 

Polyphenol-containing WW obtained after essential oil distillation of *R. damascena* was also proven to exert dose-dependent antiproliferative activity on immortalized human keratinocytes [4]. An interesting strategy for the processing of wastewater from the distillation of rose oil and the use of the contained substances was proposed by Rusanov et al. (2014); the authors, in the first stage in their research, determined the phytochemical profile of the wastewater from the distillation of rose oil from *Rosa damascena* Mill; next, they demonstrated that the RF20- (SP-207) and F (IV) subfractions isolated by them significantly inhibit cell proliferation and migration of HaCaT cells [21]. Reference is made in the scientific literature to the approaches developed for the study and valorization of wastewater—mainly from the distillation of *Rosa damascena* Mill. However, worldwide, rose oil production is based on several species: *Rosa damascena* Mill., *Rosa gallica* L., *Rosa centifolia* L., and *Rosa alba* L. [22,23]. The scarcity of studies on the composition and biological activities of the products derived from these roses, as well as the lack of data on their pharmacological and genotoxic potential, present a real challenge for scientists, and the opportunity to discover new substances with valuable qualities. 

The aim of the present study was to determine in vitro the chromatographic profiles, redox-modulating capacity, and antineoplastic activity of WWs obtained via the distillation of essential oils from the Bulgarian *Rosa alba*, *Rosa damascena*, *Rosa gallica*, and *Rosa centifolia*.

**Table 1:** Main compounds of wastewaters from *Rosa damascena* Mill., *Rosa alba* L., *Rosa gallica* L., and *Rosa centifolia* L., obtained by UHPLC-HRMS/MS analysis with clearly expressed healing, antineoplastic, and antioxidant effects.

Compounds	Relаtive Content (%) ^1^	Activity	Observations	References
Gallic acid	3.85–9.28	Anticancer Antioxidant	Inhibits cell proliferation, reduces cell viability and induces apoptosis and ferroptosis Free radical scavenger and metal chelator	[24,25,26,27,28,29,30,31,32,33,34] [28,29,31,33,34,35]
Protocatechuic acid	0.01–0.8	Anticancer Antioxidant	Inhibits cancer cell metastasis Induces cell cycle arrest and apoptosis through multiple signaling pathways from the mitogen-activated protein kinase Reduces (Fe^3+^), reducеs (Cu^2+^), scavenges superoxide anion radicals and hydroxyl radicals, chelates (Fe^2+^) and (Cu^2+^)	[36] [37,38]
Corilagin	0.23–0.45	Anti-tumor Antioxidant	Affects the signaling pathways of tumor cells; induces apoptosis Decreases malondialdehyde levels; restores the superoxide dismutase and glutathione activity; elevates the Nrf2 and heme oxygenase-1 levels in rat cerebral ischemia	[39,40,41] [39]
Proanthocyanin B2	0.01–0.75	Antineoplastic Antioxidant	Inhibits proliferation and induces apoptosis of osteosarcoma cells Reduces oxidative stress in human granulosa cells	[42,43] [44]
Catechin	0.4–5.16	Anticancer Antioxidant	Inhibits cancer cell proliferation Scavenges free radicals and retards extracellular matrix degradation induced by ultraviolet (UV) radiation and pollution	[45] [45]
Chlorogenic acid	<0.01	Anticancer Antioxidant	Serves as chemosensitizer in suppressing tumor growth through a metabolic pathway Activates ERK1/2 and inhibits proliferation of osteosarcoma cells Takes part in the control of oxidative and inflammatory stress conditions; protects DNA against oxidative damage	[46,47] [46,48]
Epicatechin	0.01–0.35	Anticancer Antioxidant	Suppress tumor cell growth Protects the bovine spermatozoa subjected to induced oxidative stress	[49] [50]
Ellagic acid	10.98–16.88	Anticancer Antioxidant	Inhibits the proliferation of prostate cancer cells; enhances the antitumor efficacy of bevacizumab in an in vitro glioblastoma model Radical scavenging activity—good scavenger of peroxynitrite	[51,52,53,54,55,56,57,58,59,60]
Rutin	<0.01	Anticancer Antioxidant	Anticancer activity in combination with ionic liquids in renal cells; regulation of different cellular signaling pathways Inhibits lipid peroxidation, xanthine oxidase, H_2_O_2_ generation, and lactate dehydrogenase	[61,62,63,64,65,66,67] [65,66,67,68]
Isoquercetin	0.43–5.98	Anticancer Antioxidant	Serves as adjunct therapy in patients with kidney cancer; inhibits bladder cancer cells; antineoplastic activity; Radical scavenging effect	[61,69,70,71,72,73]
Avicularin	0.01–5.18	Anticancer Antioxidant	Ameliorates human hepatocellular carcinoma via the regulation of NF-κB/COX-2/PPAR-γ activities; antineoplastic activity; DPPH and OH radical scavenging effect Shows protective effect against oxidative stress induced by hydrogen peroxide by inhibiting the formation of reactive oxygen species, reducing lipid peroxidation and cell death	[74,75,76] [74,75]
Quercetin	0.16–1.25	Anticancer Antioxidant	Antagonizes the cytotoxic effects of antineoplastic drugs in ovarian cancer; enhances the antiproliferative activity of cis-diamminedichloroplatinum(II); ribavirin and quercetin synergistically downregulate signal transduction, and are cytotoxic in human ovarian carcinoma cells; inhibits neck cancer; synergizes with 2-methoxyestradiol, inhibiting cell growth and inducing apoptosis in human prostate cancer cells; Scavenges intracellular free radicals	[77,78,79,80,81] [82,83,84]
Kaempferol	0.04–0.56	Anticancer Antioxidant	Anticancer potential on head and neck cancers; regulates apoptosis in diverse cancer cell models; antineoplastic activity; inhibits experimental hepatocarcinogenesis Inhibits lipid peroxidation and normalizes activities of antioxidant enzymes; radical scavenging effect	[78,85,86,87] [87,88]

## 2. Materials and Methods

### 2.1. Preparation of Wastewater from the Industrial Cycle of Water–Steam Distillation of Rose Oil

**Figure 1:**
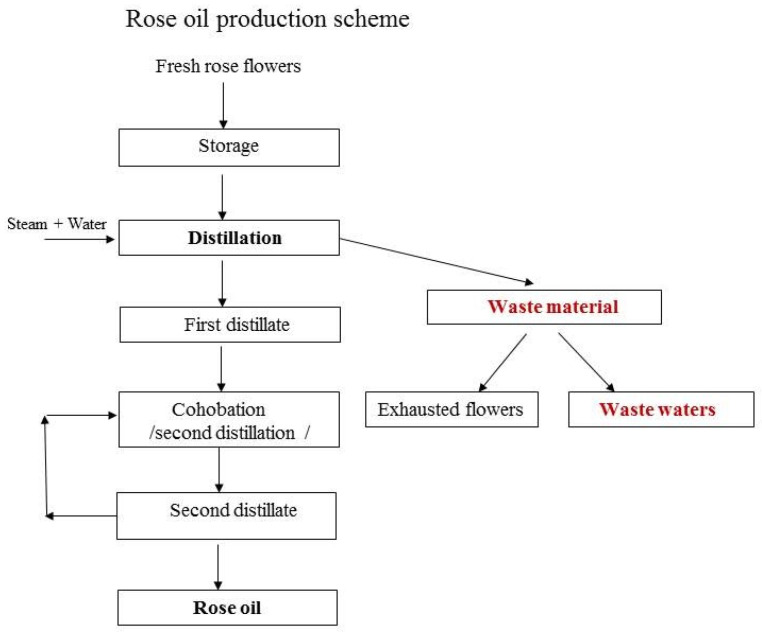
Rose oil production scheme and collection of wastewater material.

The WWs were collected after the distillation of rose flowers, as illustrated in Figure 1. The semi-industrial installation of the Institute for Roses was used. The process parameters were as follows: raw material 8–10 kg; hydromodule 1:4; flow rate 16–20 mL/min; duration 150 min. The WWs were sealed and stored in a cool place until the next stage of the investigations.

### 2.2. LC-MS of Wastewater of Rosa damascena Mill., Rosa alba L., Rosa gallica L., and Rosa centifolia L.

#### 2.2.1. Sample Preparations

The samples of WWs resulting from the steam distillation of rose flowers were prepared as follows: One milliliter of each sample was centrifuged at 14,000 rpm, and was subjected to solid-phase extraction (SPE) on Strata^®^ C18-E (55 µm, 70 Å, 200 mg, Phenomenex) cartridges, washed with distilled water and, finally, analytes were recovered with 6 mL methanol/water (80:20) mixtures. The final volume was adjusted to 10 mL.

#### 2.2.2. Chromatographic Separation and Mass Spectrometric Conditions

UHPLC-HRMS analysis was performed using a Thermo Scientific Dionex Ultimate 3000 RSLC (Germering, Germany) consisting of an SRD-3600 6-channel degasser, HPG-3400RS high-pressure gradient pump, WPS-3000TRS autosampler, and TCC-3000RS column compartment coupled with a Thermo Scientific Q Exactive Plus (Bremen, Germany) mass spectrometer. UHPLC separations were performed on a Kromasil Eternity XT C18 column (AkzoNobel, Angered, Sweden) (2.1 × 100 mm, 1.8 μm) equipped with a SecurityGuard ULTRA UHPLC EVO C18 precolumn (Phenomenex, CA, USA) at 40 °C. Each chromatographic run was carried out with a binary mobile phase consisting of water containing 0.1% (*v/v*) formic acid (A) and acetonitrile along with 0.1% (*v/v*) formic acid (B). A gradient program was used as follows: 0–1 min, 5% B; 1–25 min, 5–30% B; 25–30 min, 30–40% B; 30–32.5 min, 40–95% B; 32.5–34.5 min, 95% B. The flow rate was 0.3 mL.min^−1^ and the sample injection volume was 2 µL. The system was conditioned for 4.5 min before injection. The operating conditions for the HESI source used in a negative ionization mode were as follows: −2.5 kV spray voltage, 320 °C capillary and probe heater temperature, sheath gas flow rate of 38 a.u., auxiliary gas flow of 12 a.u. (a.u. refers to arbitrary values set by the Exactive Tune software), and S-Lens RF level of 50.00. Nitrogen was used for sample nebulization, and as the collision gas in HCD cells. Top5 was used for MS experiments, where in full MS mode the resolution, automatic gain control (AGC) target, maximum injection time (IT), and mass range were 70,000 (at m/z 200), 1e6, 80 ms, and m/z 100–1500, respectively, while ddMS2 conditions were set to resolution 17,500 (at m/z 200), AGC target 1e5, max. IT 50 ms, isolation window 2.0 m/z, and stepped normalized collision energy (NCE) of 20, 40, and 70. Xcalibur (Thermo Fisher Scientific) ver. 4.0 and FreeStyle (Thermo Fisher Scientific) ver. 1.8 SP1 were used for data acquisition and processing, respectively.

#### 2.2.3. Determination of Tannins, Flavonoids, and Total Polyphenols

The quantity of tannins and flavonoids was established according to the European Pharmacopoeia method [89,90], and expressed as mg/mL of pyrogallol equivalents for tannins, and mg/mL of hyperoside equivalents for flavonoids in rose WW. The Folin–Ciocalteu method [91], with some modifications, was used for measurement of the total phenolic content. The results were calculated as gallic acid equivalents per 1 mL of WW (mg GAE/ mL WW), based on a standard curve of gallic acid.

### 2.3. Cell Lines and Culture Conditions

In the present work, three human tumor cell lines were used—HEP-G2 (hepatocellular adenocarcinoma; the cell line retains a lot of hepatocyte-related features in the proteome, and a high degree of morphological and functional differentiation) [92,93,94,95,96], and the skin cancer lines A-375 (malignant melanoma) and A-431 (non-melanoma epidermoid squamous skin carcinoma). As a model for non-tumorigenic “normal and healthy” cells, the human skin cell line HaCaT (immortalized keratinocytes) was used. The skin cell lines were purchased from CLS Cell Lines Service (GmbH, Eppelheim, Germany), and HEP-G2 cells were obtained from DSMZ (Deutsche Sammlung von Mikroorganismen und Zellkulturen, German Collection of Microorganisms and Cell Cultures GmbH, Braunschweig, Germany). Maintenance of cells followed the protocols described previously in [97].

### 2.4. Cell Viability Assay

MTT assay was executed according to Annex C, ISO 10993-5 (ISO 10993-5:2009) for the evaluation of cytotoxic activity, as previously described [97], but there was exposure after treatment for 24 and 48 h in addition to the incubation for 72 h.

### 2.5. Mathematical Modelling of Cytotoxic Effects and Redox-Modulating Capacities of Wastewaters

The median individual effects (median inhibitory concentrations, IC_50_) of the WWs obtained from the four rose species were calculated by adapting an algorithm published elsewhere [98]. The theory of Chou and Talalay was applied, with some modifications [99]. Specifically, we coded a non-linear identification procedure in the MAPLE^®^ software based on the weighted least squares statistical criterion as an objective function of the search. To minimize the sum of weighted squares and to find the estimates of constant values, we applied a numerical optimization algorithm. The median dose model was used to obtain the “*IC_50_*” and “*m*”, as presented in Equation (1):

(1) FaFu=(DoseDm)m

where *F_a_* stands for affected fraction; *F_u_* stands for unaffected fraction *(1 − F_a_) = F_u_*; *Dose* is the applied drug concentration; *D_m_* represents the median-effect dose (in our case *D_m_ = IC_50_*), and *m* is the slope of the median-effect plot. Quantitative evaluation of effects (IC_50_) was supported by the values of the correlation coefficient R obtained for every experimental dataset. The quantitative evaluation of the redox-modulating capacities of the four wastewaters was performed using the same mathematical model. The half-maximal effective concentrations were presented as EC_50_ on the graphs.

### 2.6. Detection of Apoptosis with Annexin V

In early apoptotic cells, the membrane phospholipid phosphatidylserine (PS) is translocated from the inner to the outer leaflet of the plasma membrane, thereby exposing PS to the external cellular environment. Annexin V as a phospholipid-binding protein has a high affinity for PS, and binds to cells with exposed PS. Annexin V conjugated to the fluorochrome FITC was used in this assay to assess apoptosis. Cell suspensions (50 μL) of the same density as for the МТТ assay were seeded in the wells of a 96-well microplate for fluorescence-based assays, followed by standard 24 h incubation at 37 °C. Then, cells were treated with WWs from *R. damascena* and *R. centifolia*—doses of 1/2IC_50_ or IC_50_ (for 72 h) for the tumor cell lines, or with the 72 h IC_50_ dose of the skin cancer lines for HaCaT and HEP-G2 and exposed for 48 h. Then, cells were washed twice with cold PBS and covered with Annexin V binding buffer (component no. 51-66121E) (1 mL per 1 × 10^6^ cells were used) and FITC Annexin V (component no. 51-65874X) (5 μL for every 100 μL buffer was used). Both components were purchased from BD Biosciences (Franklin Lakes, NJ, USA). Unstained controls remained for blanks. Plates were incubated for 15 min at 25 °C in the dark, and the volume in the wells was also 5× diluted with binding buffer. Plates were read using a fluorometer (Bio-Tek Instruments Inc., Winooski, VT, USA) at 485/528 nm (excitation/emission).

### 2.7. Caspase Activity Assay

The activity of caspase-3 and -7 was evaluated after treatment of the four cell lines with WWs from *R. damascena* and *R. centifolia* for 48 h. Briefly, cell suspensions were seeded in the same quantity and density as for the МТТ assay. After 24 h of incubation, cells were treated with concentrations ranging between 1/2IC_50_ and 1.5IC_50_ for 48 h. The results were analyzed in 96-well microplates using the Apo-Glo™ assay (Promega, Germany) according to the manufacturer’s recommendations. The luminescence was measured using a luminometer (Bio-Tek Instruments Inc., VT, USA) and expressed as percentage of the untreated control.

### 2.8. Detection of Intracellular Reactive Oxygen Species Generation

The generation of reactive oxygen species (ROS) was determined using the Fluorometric Intracellular ROS Kit (#MAK143, Sigma^®^ Life Science, Darmstadt, Germany), following the manufacturer’s protocol. Cells were prepared in the same way and in the same density as for the previous assays. The results were measured in a 96-well plate (half area, transparent bottom, black walls) after 6 h of exposure to WWs from *R. damascena* and *R. centifolia*. The fluorometric reaction product proportional to the amount of ROS present was measured at λ_ex_ = 490/λ_em_ = 520 nm (Bio-Tek Instruments Inc., Winooski, VT, USA).

### 2.9. Induction of Cytochrome P450 3A4 (CYP3A4) In Vitro

The concentration of human CYP3A4 (in ng/mL) in HaCaT and HEP-G2 cell lines after 24 h of exposure to doses of IC_50_ and 2IC_50_ (for 72 h) of the most promising WWs (from *R. damascena* and *R. centifolia*) was determined using the Human Cytochrome P450 3A4 (CYP3A4) ELISA Kit (Catalog No: DL-CYP3A4-Hu, DLdevelop, Kelowna, BC, Canada), according to the manufacturer’s instructions. This test is a sandwich enzyme immunoassay based on the binding of CYP3A4 in standards and samples to a biotin-conjugated antibody preparation specific to CYP3A4. The binding of biotin to avidin conjugated to horseradish peroxidase ensures that after the addition of TMB substrate solution, only those microtiter plate wells that contain CYP3A4, biotin-conjugated antibody, and enzyme-conjugated avidin exhibit a change in color. The reaction is terminated by the addition of sulfuric acid solution, and the plate is read at 450 nm. The concentration of CYP3A4 is determined by comparing the O.D. of the samples to the standard curve.

### 2.10. Redox-Modulating Capacity of Wastewater from the Industrial Cycle of Water–Steam Distillation of Rose Oil

#### 2.10.1. Ferric-Reducing Antioxidant Power (FRAP)

FRAP assay was performed as described in [100], with light modifications. This method is based on the reduction of Fe(III) ions to Fe(II) at low pH, if the sample contains reductant (antioxidant). Thereby, the colorless complex Fe(III)–(TPTZ) 2,3,5-triphenyltetrazolium chloride turns into the blue-stained Fe(II)–TPZ complex. The analysis was performed as follows: Solutions: (1) 0.03 M acetate buffer, pH 3.6; (2) 1 mM TPTZ (2,4,6-tripyridyl-s-triazine, in 40 mM HCl); (3) 1.5 mM FeCl_3_.6H_2_O. The thus-prepared solutions were mixed in the following ratio: 10 parts 0.03 M acetate buffer (1): 1 part 1 mM TPTZ (2): 20 parts 1.5 mM FeCl_3_ (3). To 1.5 mL of the reaction mixture, 50 µL of the sample was added; blank—reaction mixture + 50 µL of H_2_O was used instead of the sample. Incubation for 4 min. at 37 °C followed. Absorption was measured at 593 nm. The results were expressed as mmol Trolox equivalents per mL of WW.

#### 2.10.2. Cupric-Reducing Antioxidant Capacity (CUPRAC) Assay

The assay described in [101] was adapted. The CUPRAC reaction of Cu(II)–neocuproine complex with antioxidants results in a change from blue to yellow/orange due to Cu(I)–neocuproine chelate (λmax = 450 nm). Solutions: (1) 10mM CuCl_2_ in dd H_2_O; (2) 1.0 M ammonium acetate buffer; pH7; (3) 7.5 mM neocuproine (NC) in 96% ethanol. The common reaction mixture was prepared in the following arrangement: 1 part Cu (II) (1): 1 part NC (3): 1 part buffer (2). In Eppendorf safe-lock tubes X mL of each tested substance was added, and the volume was brought to 0.550 mL by adding H_2_O. Next, 1.5 mL of the common reaction mixture was added. After incubation at 50 °C for 20 min, the absorption was read at 450 nm against a blank sample (1.5 mL of common reaction mixture was added to 0.550 mL of H_2_O). Standard curves were prepared with Trolox in different concentrations, ranging from 0.1 mmol to 1 mmol, and results were expressed as mmol Trolox equivalents per 1 mL of WW.

#### 2.10.3. Fe (II)-Chelating Assay

Iron(II) ions react with ferrozine to form a pink complex with a maximum absorption at 562 nm. Therefore, the presence of a chelating agent in the reaction medium will reduce the measured absorbance. Procedure: 0.2 mL of sample solution, 0.74 mL of 0.1 M sodium/acetate buffer (pH 5.23), and 0.02 mL of 2 mM solution of ferrous sulfate in 0.2 M hydrochloric acid were mixed for 10–15 s. Then, 0.04 mL of 5 mM ferrozine solution (mw: 492.46 g) was added, and the absorbance was measured after keeping it in the dark for 10 min. The Fe(II)-chelating capacity of the tested substance was determined using the following formula:

Activity (%) = 100 (Ac − As)/(Ac),

where Ac is the absorbance of the blank probe, containing 200 µL of sodium/acetate buffer instead of the sample, while As is the absorbance of the sample solution [102].

### 2.11. Statistical Analysis

The experiments for determination of the median inhibitory concentrations were performed three times, wherein each concentration was repeated four times. Samples and standards (when used) for the tests for the induction of cytochrome P450 3A4, as well as the Annexin V, caspase-3/7, and ROS tests, were carried out in duplicate. IC_50_ values expressed as µg/mL of polyphenols were calculated based on the determined IC_50_ as a percentage of WW (*v/v*) and the previously measured contents of polyphenols (mg/mL) of each WW. The selectivity index (SI) represents the IC_50_ value of an agent for the non-tumorigenic cell lines HaCaT or HEP-G2 (though a cancer line, the latter retains numerous hepatocyte-related features) divided by the IC_50_ value of a tumor cell line or the average IC_50_ value of several tumor lines. The statistical evaluation of the data and the comparison between the treated groups were performed via two-way ANOVA using the GraphPad Prism software (version 6.01 for Windows, GraphPad Software Inc., San Diego, CA, USA). Statistical processing set *p* ≤ 0.05 as the significance level.

## 3. Results

### 3.1. Chromatographic Profile and Content of Tannins, Flavonoids, and Total Polyphenols

Chromatographic analysis showed that the WWs from the four roses contained many of the same ingredients—gallic acid, glucogallin, catechin, chlorogenic acid, brevifolincarboxylic acid, epicatechin, ellagic acid, hyperoside, miquelianin, avicularin, kaempferol, and their derivatives (see Table S1 in Supplementary Materials). They exhibit very good biological activities, such as antineoplastic, antitumor, radical scavenging, and redox-modulating effects (Table 1).

The data from the quantitative analysis of tannins, flavonoids, and total polyphenols are shown in Table 2. The highest level of tannins was found in WW from *R. centifolia* (2.47 ± 0.05 mg/mL), followed by *R. alba* (2.16 ± 0.35 mg/mL). The total flavonoid content expressed as hyperoside varied from 0.37 to 1.14 mg/mL in the rose WWs. *R. damascena* and *R. alba* WWs demonstrated the highest quantities of flavonoids (1.14 ± 0.01 mg/mL and 1.00 ± 0.01 mg/mL, respectively). The content of total polyphenols was highest in *R. centifolia* (7.8 mg/mL GAE), and the lowest was the WW from *R. damascena* (7.2. mg/mL GAE).

### 3.2. Cytotoxicity of Wastewaters

**Figure 2:**
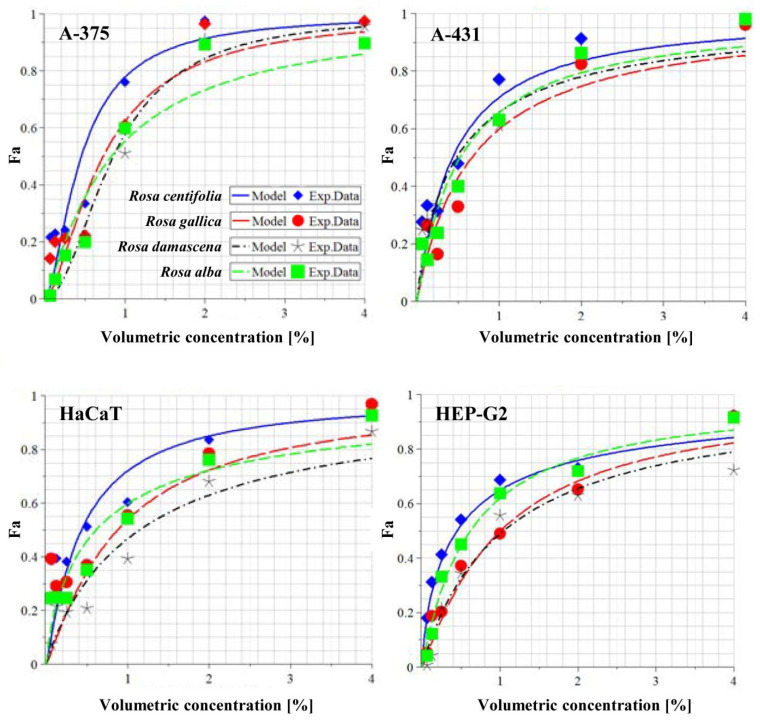
Cytotoxic activity of wastewaters from *Rosa* spp. on non-tumorigenic and tumorigenic cell lines—curve model. **Legend:** HEP-G2: liver adenocarcinoma (stage I); HaCaT: normal human keratinocytes; A-375: malignant melanoma; A-431: epidermoid carcinoma of the skin; Fa: antiproliferative effect (expressed as fraction of the untreated control). The curves describing the experimental data are presented on the plots with different types of lines and colors. The experimental data are presented as points with different shapes and colors corresponding to the color of the respective curve.

WWs from the four roses did not exert significant cytotoxic effects on the chosen human cancer and normal cell lines (Table 3, Figure 2). The median inhibitory concentrations of the WWs are presented as volumetric concentrations (%) in Table 3. In addition, the corresponding total polyphenolic contents determined as gallic acid equivalents (Table 2) are given in brackets. The lowest median inhibitory concentration (IC_50_) was of *R. centifolia* (34–35 µg GAE/mL) for 72 h for all cell lines. This is not an outstanding effect according to the National Cancer Institute guidelines, which determine that a promising extract is one with an IC_50_ < 20 µg/mL [103]. The highest IC_50_ value for the same incubation time was 83 µg GAE/mL for *R. damascena* and HaCaT cells. As usual, IC_50_ values became lower with increasing time of exposure, and for 24 h they varied between 54 and 261 µg GAE/mL. It is important to note that for the 24 h exposure, invariably, the most resistant cell line (i.e., with the highest IC_50_ values) was HEP-G2, followed by HaCaT, A-431, and A-375 (Supplementary Table S2). For 48 h, this pattern was maintained, with slight exceptions (Supplementary Table S3), while the 72 h exposure time equalized the differences to a great extent.

Therefore, the WWs at 72 h already did not have much selectivity towards cancer cell lines. The best selectivity at that incubation time was observed for *R. damascena*, which was the only WW that had a selectivity index of over 2 towards the non-melanoma skin cancer cells A-431 (compared to both HaCaT and HEP-G2 cells) (Table 4). An SI over 2 or 3 is considered a promising value in practice. Therefore, the WW from *R. damascena* has a moderate anticancer activity on non-melanoma skin cancer (IC_50_ of 35 µg/mL polyphenols), and a good toxicological safety profile.

### 3.3. Detection of Apoptosis by Annexin V and Caspase 3/7 Activity

**Figure 3:**
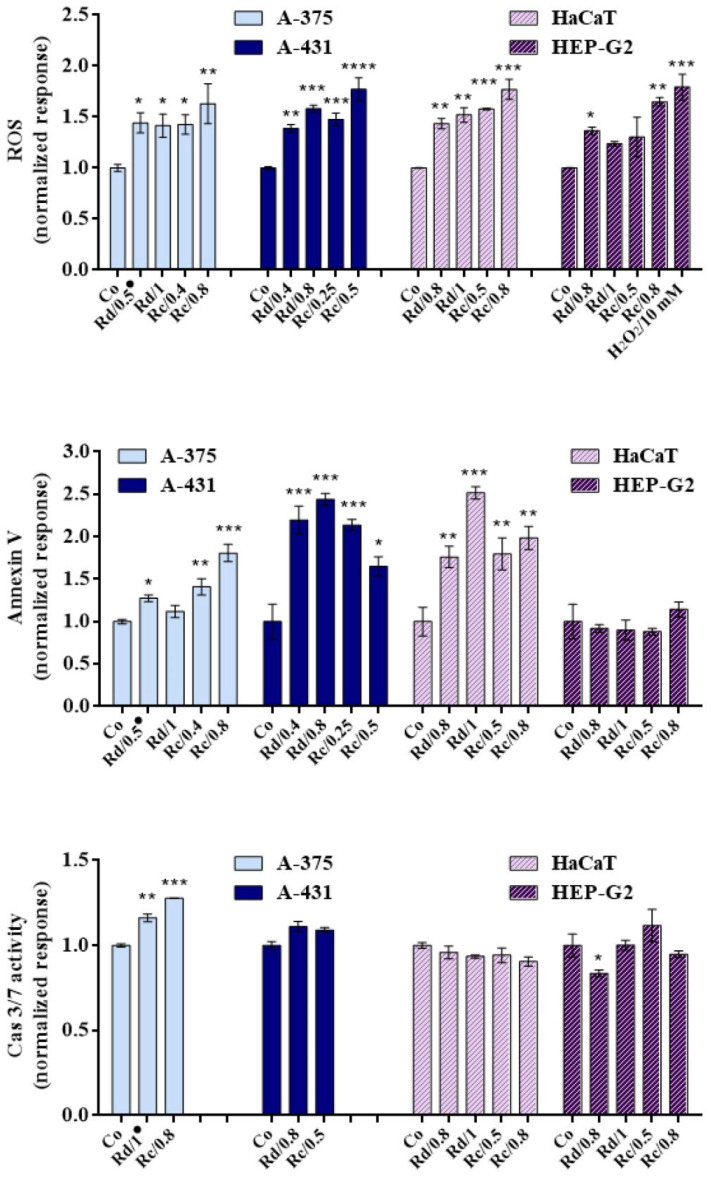
Generation of reactive oxygen species and induction of apoptosis after treatment of tumorigenic and non-tumorigenic cell lines with wastewater from two *Rosa* spp. for 48 h. Legend: Co: control; Rd: *R. damascena* Mill.; Rc: *R. centifolia* L.; ^●^: volumetric concentration (%). Significant deviations from the untreated controls are denoted with asterisks (* for *p* < 0.05, ** for *p* < 0.01, *** for *p* < 0.001, **** for *p* < 0.0001).

As we can see from the results presented in Figure 3, the WWs from *R. damascena* and *R. centifolia* induced apoptosis in all cell lines except for HEP-G2, judging by the rise in O.D values of Annexin-V-treated samples in comparison to controls. Apoptosis was induced to different extents in the A-375, A-431, and HaCaT cell lines, the most pronounced effect being in A-431 cells. Caspases cause most of the visible changes that characterize apoptotic cell death [104]. In parallel, a slight induction of caspase-3/7 was observed in the tumorigenic A-375 and A-431 cells, in contrast to the non-tumorigenic HaCaT and HEP-G2 cells. The concentrations applied in the non-tumorigenic cell lines represent the IC_50_ values estimated for the tumorigenic cells. In this way, we compare the effects of the same concentrations in both cell types. The activation of caspase-3/7 was more pronounced in A-375 cells than in A-431. No significant changes in the caspase-3/7 activity were observed in HaCaT and HEP-G2 cells.

### 3.4. Detection of Intracellular ROS Generation

The results showed a strong induction of ROS in all cell lines. Treatment with the WWs was not selective, and induced an increase in the intracellular ROS in both the tumorigenic and non-tumorigenic cell lines.

### 3.5. Effects of Rose Wastewaters on the Expression of the Enzyme CYP3A4

**Figure 4:**
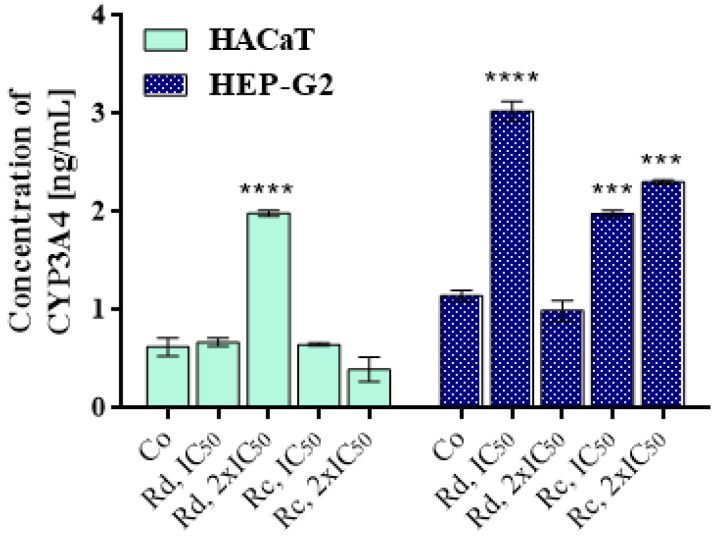
Effects of wastewaters obtained from *Rosa damascena* Mill. and *Rosa centifolia* L. on the concentration of the enzyme CYP3A4. Legend: Co: control; Rd: *R. damascena* Mill.; Rc: *R. centifolia* L.; IC_50_: median inhibitory concentration for 72 h (see Table 3). Significant deviations from the untreated controls are denoted with asterisks (*** for *p* < 0.001, **** for *p* < 0.0001).

After 24 h of exposure to the WWs, there was an increase in the concentration of CYP3A4 (induction)—to varying extents—in almost all of the treated samples (Figure 4, CYP 3A4). Notably, different doses of the WW from *R. damascena* showed a statistically significant response in both cell lines, but the response was inverse. The dose of 2IC_50_ induced CYP3A4 more than threefold in HaCaT cells, but the IC_50_ dose raised it only slightly. Meanwhile, in HEP-G2 cells, the dose of IC_50_ induced an almost threefold rise, while 2IC_50_ actually inhibited it slightly. The two doses of the WW from *R. centifolia* did not show such a dramatic difference. In HaCaT cells, the response differed little from the control (2IC_50_ reducing it by about 1/3) while, in contrast, both doses induced the enzyme significantly in HEP-G2 cells.

### 3.6. Redox-Modulating Capacity of Rose Wastеwaters

**Figure 5:**
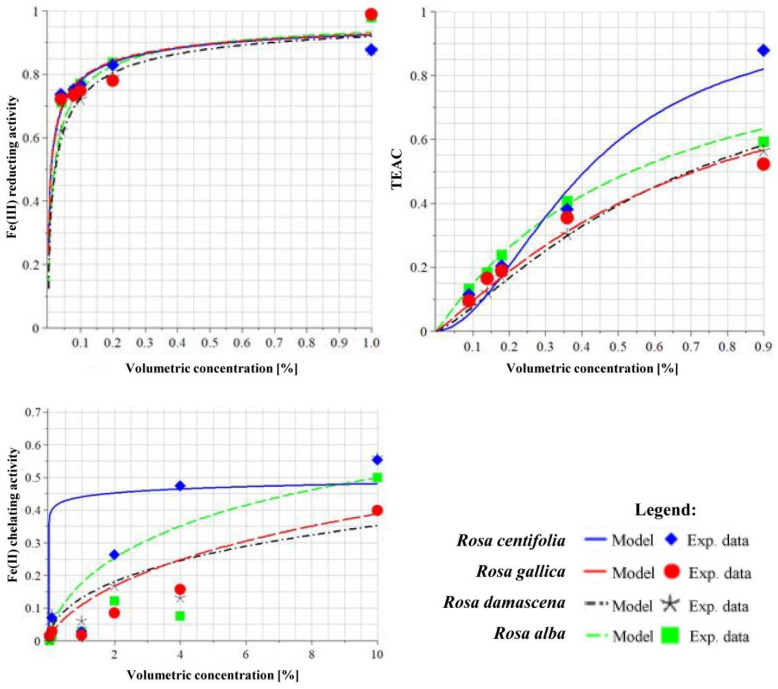
Quantitative evaluation through IC_50_ values of the redox-modulating capacity of wastewater samples, using a nonlinear median effect model.

The ability of WWs to reduce Fe(III) ions was determined via the FRAP method, and their activities were compared. Trolox was used as a reference substance, the maximum activity of which was assumed to be 1, and the activities of the test samples were calculated against it. Analyzing the data shown in Table 5 and Figure 5, the four samples showed almost equivalent Fe(III)-reducing activity, but the most active was WW from *R. damascena*. The most pronounced metal-chelating action was shown by *R. gallica* (rich in gallic acid and ellagic acid) and *R. alba* (rich in ellagic acid) wastewaters. The strongest action as a reducer of Cu(II) was shown by *R. centifolia* (rich in tannins, kaempferol, and its derivatives) and *R. alba* (rich in ellagic acid) WWs. All of the WWs showed a well-defined concentration–effect relationship.

## 4. Discussion

To the best of our knowledge, this is the first systematic study on the redox-modulating capacity and antitumor activity of rose wastewaters. It is primarily the essential oils and the organic solvent extracts from the roses or their petals that exert cytotoxic activity towards cancer cell lines, and this is valid also for *R. damascena* and its variety the Taif rose. As described in our recent review [105] regarding IC_50_, an isoprenylated aurone (IC_50_ on acute myeloid leukemia NB4 cells 4.8 μM and on neuroblastoma SHSY5Y cells 3.4 μM) from *R. damascena* and a crude methanol extract and its aqueous fraction from Taif rose (IC_50_ of 9 and 8 μg/mL on hepatocellular carcinoma cells, respectively) [106,107] were the most active agents. Essential oils, and some organic solvent extracts and their fractions, of the aforementioned roses also have low-to-significant cytotoxic effects on normal cell lines.

There are diverse data on the water-soluble compounds from rose flowers:

- The water-soluble phase of the essential oils turned out to be a potent growth factor for gastric (MKN45) and colon (SW742) cancer cell lines, and even more so for normal fibroblasts [108,109];
- The water extract of petals was not cytotoxic to murine Ehrlich ascites carcinoma cells and peripheral blood leukocytes [110];
- Infusions from petals of *R. damascena* “Alexandria” and *R. gallica* “Francesa” drafted in *R. canina* had IC_50_ values for breast (MCF-7), HEP-G2, and lung (NCI-H460) tumor cell lines of 377, 315, and >400 μg/mL, respectively [105,111].

There are many enzymatic sources of reactive oxygen species (ROS) in living organisms, including CYP enzymes, which contribute to the balance of cell oxidation–reduction [112,113,114]. Disruption of normal redox balance leads to oxidative stress, and is involved in a number of disease processes, including carcinogenesis [115]. Normally, ROS perform a biological function, but overproduction of ROS causes cell damage through modifications of lipids, nucleic acids, and proteins, as well as triggering apoptosis. CYP enzymes are known to be a superfamily of monooxygenases, and many of them are responsible for the detoxification of xenobiotics. CYP enzymes are universal catalysts for a wide range of biochemical reactions, but are well known for their role in substrate oxidation [116]. Due to their oxidizing capacity, CYP enzymes play an important role in the metabolism of phase I drugs and xenobiotics, increasing the polarity of the substrates and promoting their excretion. CYP3A4 is the CYP isoform with the lowest substrate specificity (it metabolizes more xenobiotics than any other CYP isoform).

As we can see from the results presented in Figure 3, there was a strong induction of ROS and translocation of phosphatidylserine from the inner to the outer cell membrane in the malignant cell lines A-375 and A-431, in combination with caspase-3/7 induction. It is obvious that the tested WWs exert antineoplastic effects on both cell lines by inducing apoptosis. In the mammalian cells, there are two major apoptotic pathways: (1) the death receptor pathway, and (2) the mitochondrial pathway. Both pathways include the activation of caspase-3/7 as the main executor caspases. Caspases cause most of the visible changes that characterize apoptotic cell death, and can be thought of as the central executioners of the apoptotic pathways. The same pro-apoptotic concentrations of both of the tested WWs induce ROS generation in normal keratinocytes, as well as in the HEP-G2 cell line, which was used as a model for investigation of liver toxicity. Translocation of phosphatidylserine occurred only in the HaCaT keratinocytes, and there was no caspase-3/7 activation in these cell lines, in contrast to A-375 and A-431. The cytotoxicity assay showed higher IC_50_ values for the HaCaT and HEP-G2 cells; thus, the results from the caspase induction assay correlate directly with the results of the MTT assay. In addition, the lower induction of ROS by *R. damascena* WW in HEP-G2 cells at 48 h corresponds to the lower level of cytotoxicity in comparison to the WW of *R. centifolia*.

The lack of induction of apoptosis in HEP-G2 cells after a 48 h period of exposure to both WWs, as evidenced by the Annexin V test, is consistent with the cytotoxicity test. In the latter, as mentioned, HEP-G2 was invariably the most resistant cell line for the 48- and 24-hour periods of incubation. WW from *R. centifolia* increased the concentration of CYP3A4 only in HEP-G2 but not in HaCaT cells after incubation for 24 h (the MTT test confirmed that *R. centifolia* WW was the most cytotoxic to HaCaT cells for the 24 h exposure). The short-term induction of the CYP3A4 xenobiotic oxidase enzyme could cause or contribute to the resistance of the hepatic tumor cells to treatment. The effect of the WW from *R. damascena* was the induction of CYP3A4 in both cell lines, and this is supported by the results of the MTT test for 24 h, where the effect of that WW towards HaCaT cells was almost as weak as towards HEP-G2 cells.

In principle, healthy cells and tissues are characterized by low levels of reactive oxygen species (ROS) and constant reference levels of reducing equivalents. Increasing ROS above a certain “critical” level provokes genome instability and the activation of proliferation, in which normal cells begin to transform into malignant cells. To maintain optimal cellular homeostasis, the regulation of cellular redox signaling is extremely important [117]. Upon initiation of cellular apoptosis, ROS are increased by disruption of the intracellular redox homeostasis and irreversible oxidative modifications of lipids, proteins, or DNA; this, in turn, may activate oxidative-stress-induced apoptotic signaling [118]. ROS have been shown to trigger the apoptosis of cancer cells via different mechanisms, including tumor-related necrosis factor (TNF) [119,120]. 

One of the effects of overproduction of ROS is ferroptosis; this is a type of iron-dependent oxidative cell death that can be caused by a variety of factors. Ferroptosis is different from apoptosis, but is also the result of dysfunction of antioxidant defense, leading to the loss of cellular redox homeostasis [121,122]. However, ferroptosis is also characterized by elevated levels of intracellular ROS [123].

An increase in ROS in the presence of iron ions has been shown to lead to ferroptosis. It appears that the accumulation of ROS and ferroptosis can be controlled by treatment with deferoxamine as an iron chelator [124].

The FRAP and CUPRAC methods are based on a single-electron transfer mechanism. The reducing power of FRAP is based on this mechanism, and cannot detect antioxidants that act by radical quenching (H transfer). The method is based on the reduction of Fe(III) to Fe(II) [125]. The CUPRAC method is based on the reduction of Cu(II) to Cu(I) [125]. The reduction of iron and copper, as well as the formation of their complexes, is of critical importance, since when they are in a “free” form they can catalyze the production of highly toxic hydroxyl radicals [126]. 

The high metal reduction capacity in our study suggests that WWs of roses could be the first line of defense against copper toxicity, and could serve as copper chelators by sequestering the metal in a non-redox-active form. This mechanism of antioxidant activity is beneficial to living organisms due to the prevention of oxidative damage to the cellular membranes, and is essential for cell survival. According to our investigations, WWs might perform an essential detoxification function against ions of copper and iron. This function would be beneficial for maintaining metal homeostasis and protecting the function of cellular structures against the damaging effects of reactive oxygen species.

Antioxidants that have the ability to chelate and reduce iron (III) ions are potential candidates for controlling ferroptosis and its destructive effects on healthy cells.

In the context of our results, we hypothesize that the good iron-chelating properties of WWs from roses—especially those of the Damask rose—would serve as good low-cytotoxicity adjuvants in the treatment of oncogenic conditions associated with similar aspects of impaired redox homeostasis.

## 5. Conclusions

Recent years have witnessed a resurgence of global interest in herbal drugs. More and more people are turning to the use of herbal medicinal products in health care. It is high time that we revived the hidden wonders of the plant world, hidden in waste products that we have not appreciated for a long time—they must not be considered mere biological contaminants. In our work, we have studied a panel of bioactivities from wastewaters containing complex organic compounds available from the Bulgarian production of rose oil. We found that the WWs from four different *Rosa* spp. induce the executor caspase-3/7 in tumorigenic cell lines, but not in “normal” cells. The IC_50_ values for 72 h ranged between 33 and 83 µg GAE/mL of polyphenols, which is a promising result for the future development of rose products for local treatment. The radical-modulating activity of the WWs can be used, depending on the concentrations applied, for chemoprevention, or as a part of combined chemotherapy after future pharmacological investigations. The chemical components of the WWs represent a promising source of new advanced generation agents with low cytotoxicity and pleiotropic pharmacological effects, such as antineoplastic and antioxidant activity, with a novel mode of action. In our opinion, this is only a promising beginning; hence, further studies are urgently needed for screening waste products and determining their bioactivities and properties in order to understand the complete phenomena of their healing potential.

## Figures and Tables

**Table 2:** Content of tannins, flavonoids, and total polyphenols in wastewaters from the tested roses.

Wastewater	^2^ Tannins (mg/mL)	^1^ Total Flavonoids (mg/mL)	^3^ Total Polyphenols (mg/mL)
*Rosa damascena* Mill.	1.61 ± 0.05	1.14 ± 0.01	7.2 ± 0.2
*Rosa alba* L.	2.16 ± 0.35	1.00 ± 0.01	7.6 ± 0.3
*Rosa gallica* L.	1.51 ± 0.09	0.37 ± 0.02	7.7 ± 0.03
*Rosa centifolia* L.	2.47 ± 0.05	0.61 ± 0.04	7.8 ± 0.22

^1^ Calculated as hyperoside; ^2^ calculated as pyrogallol; ^3^ calculated as gallic acid equivalents (µg GAE/mL).

**Table 3:** Median inhibitory concentrations of wastewaters obtained from different rose species in non-tumorigenic and tumor cell lines after 72 h of incubation.

Cell Line	Model Parameters	WW from *R. centifolia* L.	WW from *R. gallica* L.	WW from *R. damascene* Mill.	WW from *R. alba* L.
HEP-G2	HillSlope	0.795	1.115	0.988	1.017
	IC_50_	0.45% * (=35.1 µg GAE **/mL)	1.01% (=77.77 µg GAE/mL)	1.049% (=75.53 µg GAE/mL)	0.622% (=47.27 µg GAE/mL)
	R (correlation coefficient)	0.997	0.994	0.993	0.997
HaCaT	HillSlope	1.13	1.16	0.954	0.81
	IC_50_	0.435% (=33.93 µg GAE/mL)	0.879% (=67.68 µg GAE/mL)	1.15% (=82.8 µg GAE/mL)	0.616% (=46.82 µg GAE/mL)
	R (correlation coefficient)	0.966	0.966	0.985	0.982
A-375	HillSlope	1.582	1.576	1.976	1.15
	IC_50_	0.455% (=35.49 µg GAE/mL)	0.729% (=56.13 µg GAE/mL)	0.857% (=61.7 µg GAE/mL)	0.835% (=63.46 µg GAE/mL)
	R (correlation coefficient)	0.979	0.985	0.996	0.99
A-431	HillSlope	1.062	0.991	0.893	1.01
	IC_50_	0.435% (=33.93 µg GAE/mL)	0.672% (=51.74 µg GAE/mL)	0.485% (=34.92 µg GAE/mL)	0.53% (=40.28 µg GAE/mL)
	R (correlation coefficient)	0.987	0.982	0.985	0.99

Legend: HEP-G2: liver adenocarcinoma (stage I); HaCaT: normal human keratinocytes; A-375: malignant melanoma; A-431: epidermoid carcinoma of the skin; HillSlope: slope factor or Hill slope, unitless; IC_50_: median inhibitory concentration; *: volumetric concentration in (%); **: (µg GAE/mL) = concentration of total polyphenols determined as gallic acid equivalents.

**Table 4:** Comparison of the cytotoxicity of wastewaters obtained from four different rose species on non-tumorigenic and tumorigenic cell lines, based on selectivity index values.

*Rosa* spp.	Cytotoxicity Based on IC_50_ Values	SI Values
*R. centifolia* L.	A-431 * = HaCaT * > HEP-G2 > A-375 **	SI_HaCaT/A-375_ = 0.96 SI_HaCaT/A-431_ = 1.00 SI_HEP-G2/A-375_ = 0.99 SI_HEP-G2/A-431_ = 1.03
*R. gallica* L.	A-431 * > A-375 > HaCaT > HEP-G2 **	SI_HaCaT/A-375_ = 1.21 SI_HaCaT/A-431_ = 1.31 SI_HEP-G2/A-375_ = 1.39 SI_HEP-G2/A-431_ = 1.50
*R. damascena* Mill.	A-431 * > A-375 > HEP-G2 > HaCaT **	SI_HaCaT/A-375_ = 1.34 SI_HaCaT/A-431_ = 2.37 SI_HEP-G2/A-375_ = 1.22 SI_HEP-G2/A-431_ = 2.16
*R. alba* L.	A-431 * > HaCaT > HEP-G2 > A-375 **	SI_HaCaT/A-375_ = 0.74 SI_HaCaT/A-431_ = 1.16 SI_HEP-G2/A-375_ = 0.74 SI_HEP-G2/A-431_ = 1.17

Legend: *: most sensitive cell line; **: least sensitive cell line; SI: selectivity index.

**Table 5:** Median effective concentration of redox-modulating capacity of wastewaters from four different rose species, determined by Cu(I)- and Fe(II)-chelating and Fe(III)-reducing activity.

Redox and Chelating Activity		WW from *R. centifolia* L.	WW from *R. gallica* L.	WW from *R. damascene* Mill.	WW from *R. alba* L.
Method	Model Parameters				
**TEAC_CUPRAC_**	HillSlope	1.916	1.16	1.295	1.055
	EC_50_	0.409 *	0.714 *	0.699 *	0.538 *
	R (correlation coefficient)	0.994	0.995	0.9998	0.998
**FRAP**	HillSlope	0.538	0.538	0.638	0.66
	EC_50_	0.0095 *	0.009 *	0.022 *	0.019 *
	R (correlation coefficient)	0.998	0.995	0.999	0.998
**Fe (II) chelation activity**	HillSlope	0.072	0.715	0.560	0.715
	EC_50_	29.87 *	18.99 *	29.88 *	18.99 *
	R (correlation coefficient)	0.920	0.968	0.928	0.968

Legend: m: hillslope; EC: effective dose; EC_50_: median effective concentration. R: coefficient of correlation; *: volumetric concentration (% *v/v*).

## Data Availability

Data are contained within the article and Supplementary Materials.

## Supplementary Materials

The following are available online at https://www.mdpi.com/article/10.3390/antiox10101615/s1: Table S1: Detected chemical constituents in wastewaters from Rosa damascena (RDAM), R. gallica (RGAL), R. centifolia (RCEN), and R. alba (RALB) by UHPLC-HRMS/MS analysis. Table S2: Cytotoxicity 24 h. Table S3: Cytotoxicity 48 h.
