# Supplementary material for: Redox-Modulating Capacity and Antineoplastic Activity of Wastewater Obtained from the Distillation of the Essential Oils of Four Bulgarian Oil-Bearing Roses
Source: Antioxidants (Basel). 2021 Oct 14;10(10):1615. doi: 10.3390/antiox10101615 (PMC8533594; doi:10.3390/antiox10101615)
Supplement: Supplementary file 1 [file antioxidants-10-01615-s001.zip › Suppl Table 1.pdf]

Table S1 Detected chemical constituents in wastewaters from *Rosa damascena* (RDAM), *R. gallica* (RGAL), *R. centifolia* (RCEN) and *R. alba* (RALB) by UHPLC-HRMS/MS analysis.

No	Compound name	RT (min)	Found mass	Species	Formula	MS/MS (intensity)	Wastewater from			
							RDAM[1]	RGAL[1]	RCEN[1]	RALB[1]
1	Gallic acid	1.66	169.0132	$[M-H]^-$	$C_7H_5O_5$	125.023 (100)	3.85	9.28	4.57	5.63
2	Glucogallin	2.67	331.0674	$[M-H]^-$	$C_{13}H_{15}O_{10}$	169.0132 (100), 125.023 (60)	0.01	1.54	<0.01	0.1
3	Bis-galloyl-hexose	2.89	483.0781	$[M-H]^-$	$C_{20}H_{19}O_{14}$	331.0675 (30), 313.0571 (20), 169.0131 (100), 125.0231 (75)	1.41	0.02	2.09	0.6
4	Protocatechuic acid	3.25	153.0182	$[M-H]^-$	$C_7H_5O_4$	109.0280 (100)	<0.01	0.5	0.08	-
5	Bis-galloyl-hexose	3.67	483.0784	$[M-H]^-$	$C_{20}H_{19}O_{14}$	331.0668 (10), 313.0569 (70), 169.0133 (100), 125.0231 (60)	0.23	-	0.42	-
6	Bis-galloyl-hexose	3.85	483.0784	$[M-H]^-$	$C_{20}H_{19}O_{15}$	331.0671 (15), 313.0568 (20), 271.0462 (25), 211.0243 (30), 169.0132 (90), 125.0230 (70)	0.2	0.23	0.2	0.23
7	Corilagin	4.33	633.0739	$[M-H]^-$	$C_{27}H_{21}O_{18}$	481.0622 (10), 300.9991 (100), 275.0197 (20)	0.23	-	0.45	-
8	Methylgalloyl-hexoside	4.66	345.0829	$[M-H]^-$	$C_{14}H_{17}O_{10}$	183.0290 (100)	0.65	3.12	0.26	0.24

**9**	Protocatechuic acid glucoside	4.93	315.0726	$[M-H]^-$	$C_{13}H_{15}O_9$	153.0183 (100)	-	0.3	0.04	-
**10**	Proantocianidin B2	5.52	577.1357	$[M-H]^-$	$C_{30}H_{25}O_{12}$	425.0893 (10), 407.0768 (60), 425.0893 (80), 161.0232 (20), 125.0230 (100)	<0.01	0.75	0.01	-
**11**	Bis-galloyl-quinic acid	5.61	495.0785	$[M-H]^-$	$C_{21}H_{19}O_{14}$	343.0672 (100), 191.0553 (80), 169.0132 (80), 169.0132 (100), 125.0230 (40)	-	<0.01	-	0.3
**12**	Proantocianidin B-type dimer	5.92	577.1357	$[M-H]^-$	$C_{30}H_{25}O_{13}$	407.0772 (50), 289.0717 (70), 125.0231 (100)	<0.01	0.13	<0.01	0.04
**13**	HHDP-di-galloyl-glucose	5.96	785.0849	$[M-H]^-$	$C_{34}H_{25}O_{22}$	483.0774 (10), 300.9993 (100), 275.0202 (40), 249.0405 (30), 169.0133 (15), 125.0230 (10)	<0.01	-	0.72	-
**14**	Bis-galloyl-hexose	6.04	483.0785	$[M-H]^-$	$C_{20}H_{19}O_{14}$	313.0571 (20), 271.0463 (80), 211.0243 (100), 193.0135 (45), 169.0131 (80), 125.0231 (70), 107.0124 (20)	0.11	<0.01	<0.01	<0.01
**15**	Bis-galloyl-quinic acid	6.12	495.0785	$[M-H]^-$	$C_{21}H_{19}O_{14}$	343.0670 (70), 325.0572 (10), 191.0550 (15), 169.0133 (100), 125.0230 (40)	-	<0.01	-	0.3
**16**	Catechin	6.18	289.0719	$[M-H]^-$	$C_{15}H_{13}O_6$	245.0816 (50), 203.0707 (30), 179.0342 (20), 151.0389 (20), 123.0439 (40), 109.0280 (60)	0.4	5.16	0.74	2.2
**17**	Chlorogenic acid	6.70	353.0880	$[M-H]^-$	$C_{16}H_{17}O_9$	191.0554 (100), 179.0340 (10), 173.0446 (15), 135.0439 (10)	<0.01	<0.01	<0.01	<0.01
**18**	Brevifolincarboxylic acid	6.99	291.0149	$[M-H]^-$	$C_{13}H_7O_8$	247.0245 (100), 219.0293 (10), 191.0341 (20), 173.0235 (10), 145.0282 (10)	<0.01	1.08	<0.01	1.34

**19**	Tris-galoyl-hexose	7.02	635.0892	$[M-H]^-$	$C_{27}H_{23}O_{18}$	465.0671 (75), 421.0781 (10), 313.0570 (45), 169.0131 (100), 125.0231 (65)	1.67	-	2.26	-
**20**	HHDP-di-galloyl-glucose	8.29	785.0848	$[M-H]^-$	$C_{34}H_{25}O_{22}$	483.0786 (10), 300.9992 (100), 275.0202 (35), 249.0406 (25), 169.0132 (13), 125.0230 (15)	0.53	-	1.31	-
**21**	Epicatechin	8.66	289.0721	$[M-H]^-$	$C_{15}H_{13}O_6$	245.0092 (70), 217.0136 (20), 189.0184 (15), 173.0234 (20), 161.0233 (18), 145.0282 (35), 117.0332 (17)	<0.01	0.35	<0.01	<0.01
**22**	Digalloyl deoxyhexose	8.70	467.0831	$[M-H]^-$	$C_{20}H_{19}O_{13}$	423.0932 (25), 315.0726 (20), 169.0131 (40), 152.0103 (35), 125.0230 (30), 108.0202 (45)	-	-	-	0.31
**23**	Phenylethyl-hexoside-pentoside	8.89	461.1669	$[M+FA-H]^-$	$C_{20}H_{29}O_{12}$	415.1608 (100), 269.1029 (70), 169.0132 (15), 161.0446 (30), 101.0229 (40)	-	-	-	0.15
**24**	Phenylethyl-hexoside-pentoside	9.49	461.1667	$[M+FA-H]^-$	$C_{20}H_{29}O_{12}$	415.1614 (10), 191.0554 (55), 179.0552 (25), 149.0444 (80), 131.0337 (80)	2.12	-	0.48	0.49
**25**	1'-Monodecarboxyvaloneic acid dilactone	9.88	469.0051	$[M+FA-H]^-$	$C_{21}H_9O_{13}$	425.0151 (15), 299.9913 (100), 270.9887 (10)	<0.01	<0.01	<0.01	<0.01
**26**	Ellagic acid hexoside	9.92	463.0522	$[M-H]^-$	$C_{20}H_{15}O_{13}$	300.9990 (100)	-	2.41	-	-
**27**	Ellagic acid hexoside	10.46	463.0521	$[M-H]^-$	$C_{20}H_{15}O_{13}$	300.9992 (100)	<0.01	0.27	<0.01	<0.01
**28**	Phenylethyl-hexoside-pentoside	10.49	461.1666	$[M+FA-H]^-$	$C_{20}H_{29}O_{12}$	415.1609 (20), 179.0551 (20), 149.0444 (70), 131.0337 (30)	1.74	<0.01	0.05	<0.01
**29**	Quercetine-3-O-hexoside-hexoside	11.20	625.1409	$[M-H]^-$	$C_{27}H_{29}O_{17}$	300.0277 (100), 271.0247 (45), 255.0296 (20), 243.0296 (16)	-	-	-	6.96

**30**	Quercetine-3-O-hexoside-hexoside	11.42	625.1408	$[M-H]^-$	$C_{27}H_{29}O_{17}$	300.0277 (100), 271.0247 (45), 255.0296 (20), 243.0296 (16)	-	-	-	6.2
**31**	Isorhamnetin-3-O-gentiobioside	12.40	639.1573	$[M-H]^-$	$C_{28}H_{31}O_{17}$	314.0435 (60), 299.0200 (100), 271.0250 (35), 215.0345 (20)	0.32	0.75	-	0.13
**32**	Kaempferol-3-O-hexoside-hexoside	12.78	609.1463	$[M-H]^-$	$C_{27}H_{29}O_{16}$	284.0328 (100), 255.0298 (60), 227.0346 (45)	-	-	-	0.57
**33**	Ellagoyl-pentoside	12.93	433.0414	$[M-H]^-$	$C_{19}H_{13}O_{12}$	300.9991 (100), 271.0612 (10), 216.0057 (10)	<0.01	0.12	0.25	-
**34**	Kaempferol-3-O-hexoside-hexoside	13.01	609.1461	$[M-H]^-$	$C_{27}H_{29}O_{16}$	284.0327 (100), 255.0296 (60), 227.0345 (40)	-	-	-	4.63
**35**	Quercetine-3-O-pentoside-hexoside	13.29	595.1309	[M-H]-	$C_{26}H_{27}O_{16}$	300.0277 (100), 271.0247 (40), 255.0297 (20), 243.0296 (10)	-	-	-	0.53
**36**	Ellagoyl-pentoside	13.35	433.0414	$[M-H]^-$	$C_{19}H_{13}O_{12}$	299.9911 (100), 216.0060 (10)	0.09	0.09	0.06	-
**37**	Ellagic acid	13.56	300.9988	$[M-H]^-$	$C_{14}H_5O_8$	300.9990 (100), 283.9963 (90), 257.0085 (20), 245.0088 (5), 229.0138 (70), 201.0183 (80) 185.0236 (70), 173.0233 (75), 157.0283 (45), 145.0282 (75)	10.98	16.88	12.89	14.12
**38**	Rutin	13.80	609.1463	$[M-H]^-$	$C_{27}H_{29}O_{16}$	300.0279 (100), 271.0251 (50), 255.0300 (25), 243.0297 (15), 151.0024 (10)	<0.01	-	-	<0.01
**39**	Hyperoside	13.89	463.0883	$[M-H]^-$	$C_{21}H_{19}O_{12}$	300.0274 (100), 271.0251 (45), 255.0300 (20), 243.0297 (15), 151.0025 (10)	5.67	4.69	5.8	3.68
**40**	Miquelianin	13.97	477.0671	$[M-H]^-$	$C_{21}H_{17}O_{13}$	301.0356 (100), 178.9977 (15), 151.0024 (25)	<0.01	<0.01	<0.01	8.64

**41**	Isoquercitrin	14.25	463.0883	[M−H]⁻	$C_{21}H_{19}O_{12}$	300.0275 (100), 271.0252 (50), 255.0300 (25), 243.0298 (15), 151.0025 (10)	5.98	0.43	5.89	3.57
**42**	Quercetin-3-O-pentoside	15.01	433.0777	[M−H]⁻	$C_{20}H_{17}O_{11}$	300.0276 (100), 271.0247 (50), 255.0296 (20), 243.0297 (15), 151.0025 (10)	0.5	-	1.82	0.12
**43**	Quercetin-3-O-galloyl-hexoside	15.11	615.0997	[M−H]⁻	$C_{28}H_{23}O_{16}$	301.0356 (100), 193.0138 (10), 178.9976 (15), 151.0025 (30)	0.58	0.37	-	0.16
**44**	Kaempferol-3-O-pentoside-hexoside	15.20	579.1360	[M−H]⁻	$C_{26}H_{27}O_{15}$	284.0327 (100), 255.0297 (55), 227.0346 (40)	-	-	-	0.36
**45**	Kaempferol-3-O-galactoside	15.48	447.0933	[M−H]⁻	$C_{21}H_{19}O_{11}$	284.0328 (85), 255.0297 (80), 227.0346 (75)	4.63	1.6	0.95	1.33
**46**	Kaempferol-hexoside-methylpentoside	15.76	593.1514	[M−H]⁻	$C_{27}H_{29}O_{15}$	285.0404 (100), 255.0298 (45), 227.0345 (30)	2.07	-	<0.01	<0.01
**47**	Avicularin	15.78	433.0776	[M−H]⁻	$C_{20}H_{17}O_{11}$	301.0351 (100), 271.0252 (45), 255.0301 (25), 243.0298 (15), 178.9977 (10), 151.0025 (15)	<0.01	3.09	5.18	4.3
**48**	Quercetin-hexoside-methylpentoside	15.90	609.1460	[M−H]⁻	$C_{27}H_{29}O_{16}$	301.0354 (100), 271.0248 (30), 255.0298 (20), 243.0296 (10), 178.9979 (15), 151.0025 (30)	1.09	<0.01	-	-
**49**	Kaempferol-3-O-glucoside	16.24	447.0933	[M−H]⁻	$C_{21}H_{19}O_{11}$	284.0326 (78), 255.0297 (80), 227.0346 (78), 183.0444 (10)	16.53	1.02	2.98	6
**50**	Kaempferol-galloyl-hexoside	17.22	599.1047	[M−H]⁻	$C_{28}H_{23}O_{15}$	313.0568 (15), 285.0406 (100), 151.0024 (10)	1.29	-	-	0.23
**51**	Kampferol-pentoside	17.36	417.0828	[M−H]⁻	$C_{20}H_{17}O_{10}$	284.0326 (85), 255.0298 (80), 227.0346 (83), 183.0444 (10)	2.15	<0.01	0.84	<0.01

**52**	Eschweilenol A	17.49	425.0152	[M−H]⁻	$C_{20}H_9O_{11}$	299.9911 (100), 270.9887 (10), 216.0058 (10)	-	4.72	1.2	2.78
**53**	Kampferol-3-O-arabinoside	18.01	417.0828	[M−H]⁻	$C_{20}H_{17}O_{10}$	284.0325 (100), 255.0298 (95), 227.0346 (90), 183.0444 (10)	5.32	2.49	2.49	2.81
**54**	Kaempferol-hexoside-methylpentoside	18.08	593.1514	[M−H]⁻	$C_{27}H_{29}O_{15}$	285.0404 (100), 229.0504 (10)	<0.01	<0.01	-	-
**55**	Kaempferol-3-O-rhamnoside	18.78	431.0983	[M−H]⁻	$C_{21}H_{19}O_{10}$	285.0402 (100), 255.0297 (65), 227.0346 (60), 183.0445 (10)	5.4	0.81	4.6	1.24
**56**	Quercetin-3-acetyl-hexoside-methylpentoside	19.50	651.1574	[M−H]⁻	$C_{29}H_{31}O_{17}$	301.0354 (100), 271.0251 (35), 255.0299 (20), 243.0296 (10), 178.9975 (20), 151.0024 (35)	0.62	0.12	-	-
**57**	Kaempferol-galloyl-pentose	19.60	569.0943	[M−H]⁻	$C_{27}H_{21}O_{14}$	285.0404 (100), 229.0503 (10)	<0.01	-	0.05	-
**58**	Quercetin-p-coumaroyl-hexoside	20.91	609.1255	[M−H]⁻	$C_{30}H_{25}O_{14}$	463.0883 (65), 300.0275 (100), 271.0252 (50), 255.0300 (25), 243.0297 (15), 178.9975 (10), 151.0024 (15)	0.19	0.17	0.7	0.35
**59**	Quercetin	21.33	301.0355	[M−H]⁻	$C_{15}H_9O_7$	178.9977 (25), 151.0025 (70), 121.0281 (20), 107.0124 (25), 65.0017 (25)	-	0.16	1.25	0.55
**60**	Kaempferol-3-acetyl-hexoside-methylpentoside	21.76	635.1618	[M−H]⁻	$C_{29}H_{31}O_{16}$	285.0402 (100), 229.0503 (10)	0.06	<0.01	-	-
**61**	Eschweilenol B isomer	21.82	422.9996	[M−H]⁻	$C_{20}H_7O_{11}$	376.9933 (70), 311.0203 (70), 299.9909 (70), 293.0093 (65), 270.9884 (100)	<0.01	1.83	2.95	2.07
**62**	Kaempferol-galloyl-pentose	22.25	569.0941	[M−H]⁻	$C_{27}H_{21}O_{14}$	285.0405 (100), 229.0504 (10)	0.16	-	<0.01	-

**63**	Eschweilenol B isomer	22.71	422.9999	$[M-H]^-$	$C_{20}H_7O_{11}$	376.9946 (70), 311.0197 (100), 299.9907 (55), 293.0095 (85), 270.9887 (85)	<0.01	0.21	0.26	0.39
**64**	Kaempferol-p-coumaroyl-hexoside	23.17	593.1303	$[M-H]^-$	$C_{30}H_{25}O_{13}$	447.0940 (10), 285.0404 (100), 255.0299 (55), 227.0346 (45), 145.0285 (10)	2.72	0.83	1.09	1.14
**65**	Kaempferol-p-coumaroyl-hexoside	23.66	593.1305	$[M-H]^-$	$C_{30}H_{25}O_{13}$	447.0932 (10), 285.0402 (100), 255.0297 (55), 227.0346 (40), 145.0282 (10)	0.21	-	-	0.1
**66**	Kaempferol	25.57	285.0407	$[M-H]^-$	$C_{15}H_9O_6$	285.0407 (100), 255.0288 (25), 239.0343 (40), 227.0351 (30), 211.0394 (35), 185.0599 (50), 159.0438 (45), 143.0489 (60), 135.0072 (20)	0.56	-	0.2	0.04

[1] The values are calculated by normalization method and represent the percent of the area of the base peak. The raw data were processed with Thermo Scientific Freestyle software package.
