# Supplementary material for: Redox-Modulating Capacity and Antineoplastic Activity of Wastewater Obtained from the Distillation of the Essential Oils of Four Bulgarian Oil-Bearing Roses
Source: Antioxidants (Basel). 2021 Oct 14;10(10):1615. doi: 10.3390/antiox10101615 (PMC8533594; doi:10.3390/antiox10101615)
Supplement: Supplementary file 1 [file antioxidants-10-01615-s001.zip › Suppl Table 2 Cytotoxicity 24 h.pdf]

Table S2. Median inhibitory concentrations of wastewaters obtained from different Roses species in non-tumorigenic and tumorigenic cell lines after 24 h of incubation.

Roses WW / Cell line/Model parameters	***WW from Rosa centifolia***	***WW from Rosa gallica***	***WW from Rosa damascena***	***WW from Rosa alba***
**HEP-G2**				
HillSlope	1.376	3.22	2.095	1.675
IC_{50}	***3.344*/261****	***2.939/226***	***3.001/216***	***2.566/195***
R (correlation coefficient)	0.90	0.90	0.875	0.903
**HaCaT**				
HillSlope	1.28	1.304	1.122	1.319
IC_{50}	***1.56/121***	***1.656/127***	***2.84/204***	***1.85/140***
R (correlation coefficient)	0.91	0.91	0.92	0.87
**A-375**				
HillSlope	1.475	2.602	1.044	1.166
IC_{50}	***0.696/54***	***0.823/63***	***1.015/73***	***0.75/57***
R (correlation coefficient)	0.93	0.95	0.90	0.90
**A-431**				
HillSlope	0.895	1.279	1.663	1.447
IC_{50}	***0.853/67***	***1.294/100***	***1.852/133***	***1.345/102***
R (correlation coefficient)	0.90	0.91	0.95	0.94

Legend: HEP-G2 – liver adenocarcinoma (stage I); HaCaT – normal human keratinocytes; A-375 – malignant melanoma; A-431 – epidermoid carcinoma of the skin; HillSlope – Slope factor or Hill slope, unitless; IC_{50} – median inhibitory concentration; *volumetric concentration [%]; **concentration of polyphenols , GAE [µg/mL].
