# Supplementary material for: Redox-Modulating Capacity and Antineoplastic Activity of Wastewater Obtained from the Distillation of the Essential Oils of Four Bulgarian Oil-Bearing Roses
Source: Antioxidants (Basel). 2021 Oct 14;10(10):1615. doi: 10.3390/antiox10101615 (PMC8533594; doi:10.3390/antiox10101615)
Supplement: Supplementary file 1 [file antioxidants-10-01615-s001.zip › Suppl Table 3 Cytotoxicity 48 h.pdf]

Table S3. Median inhibitory concentrations of wastewaters obtained from different Roses species in non-tumorigenic and tumorigenic cell lines after 48 h of incubation.

Roses WW / Cell line/Model parameters	***WW from Rosa centifolia***	***WW from Rosa gallica***	***WW from Rosa damascena***	***WW from Rosa alba***
**HEP-G2**				
HillSlope	0.885	3.023	0.919	1.176
IC_{50}	***1.44/112***	***2.131/164***	***3.338/240***	***1.564/119***
R (correlation coefficient)	0.94	0.99	0.96	0.93
**HaCaT**				
HillSlope	0.756	0.949	1.125	0.844
IC_{50}	***0.618/48***	***0.876/67***	***1.38/99***	***0.835/63***
R (correlation coefficient)	0.90	0.92	0.90	0.89
**A-375**				
HillSlope	1.163	1.804	1.313	1.376
IC_{50}	***0.531/41***	***0.787/61***	***0.737/53***	***0.633/48***
R (correlation coefficient)	0.91	0.92	0.92	0.90
**A-431**				
HillSlope	0.941	0.901	1.185	1.138
IC_{50}	***0.457/36***	***0.615/47***	***0.905/65***	***0.71/54***
R (correlation coefficient)	0.92	0.90	0.91	0.93

Legend: HEP-G2 – liver adenocarcinoma (stage I); HaCaT – normal human keratinocytes; A-375 – malignant melanoma; A-431 – epidermoid carcinoma of the skin; HillSlope – Slope factor or Hill slope, unitless; IC_{50} – median inhibitory concentration; *volumetric concentration [%]; **concentration of polyphenols [GAE, µg/mL].
